# Open Partial Horizontal Laryngectomy as a Conservative Salvage Treatment for Laser-Recurrent Laryngeal Cancer: A Multi-Institutional Series

**DOI:** 10.3390/curroncol32010012

**Published:** 2024-12-27

**Authors:** Erika Crosetti, Andrea Borello, Andy Bertolin, Izabela Costa Santos, Marco Fantini, Giulia Arrigoni, Ilaria Bertotto, Andrea Elio Sprio, Fernando Luiz Dias, Giuseppe Rizzotto, Giovanni Succo

**Affiliations:** 1 Otorhinolaryngology Unit, San Giovanni Bosco Hospital, 10154 Torino, Italy; marcofantini8811@hotmail.it (M.F.); giulia.arrigoni@aslcittaditorino.it (G.A.); giovanni.succo@unito.it (G.S.); 2 Otorhinolaryngology Unit, Michele and Pietro Ferrero Hospital, 12060 Verduno, Italy; a.borello@unito.it; 3 Otorhinolaryngology Unit, Vittorio Veneto Hospital, AULSS2 Treviso, 31029 Vittorio Veneto, Italy; andy.bertolin@aulss2.veneto.it (A.B.); giuseppe.rizzotto@aulss2.veneto.it (G.R.); 4 Brazilian National Cancer Institute, Rio de Janeiro 20230-130, RJ, Brazil; ibellacs@yahoo.com.br (I.C.S.); fdias@inca.gov.br (F.L.D.); 5 Radiology Service, Candiolo Cancer Institute, FPO IRCCS, Candiolo, 10060 Turin, Italy; ilaria.bertotto@ircc.it; 6 Department of Research, ASOMI College of Sciences, 2080 Marsa, Malta; a.sprio@asomi-osteopatia.com; 7 Oncology Department, University of Turin, 10124 Torino, Italy

**Keywords:** laryngeal cancer, partial laryngectomy, OPHL, laryngeal preservation, laser, recurrence

## Abstract

Early-stage laryngeal cancer (T1-T2) is commonly treated with organ-preserving techniques such as transoral laser microsurgery (TOLMS) or radiation therapy (RT), both providing comparable oncological outcomes but differing in functional results. Local recurrence occurs in approximately 10% of cases, making salvage surgery a crucial therapeutic option. This multi-institutional study investigates the efficacy of open partial horizontal laryngectomy (OPHL) as a salvage treatment, following recurrent laryngeal squamous-cell carcinoma (LSCC) after failed TOLMS. This analysis includes 66 patients who underwent OPHL between 1995 and 2017, reporting favorable oncological outcomes with overall survival (OS) of 87.4%, disease-specific survival (DSS) of 93.4%, and disease-free survival (DFS) of 85.5%. A recurrence rate of 10.6% was observed post-salvage OPHL, with vascular invasion and advanced pathological staging identified as significant predictors of recurrence. OPHL emerged as an effective organ-preserving alternative to total laryngectomy (TL) in select patients, especially those with limited tumor spread and preserved laryngeal function. The study highlights the importance of careful patient selection and thorough preoperative assessment to improve outcomes, positioning OPHL as a key option in treating recurrent laryngeal cancer and offering oncological control while preserving laryngeal functions.

## 1. Introduction

Early laryngeal cancer represents a significant clinical challenge in head and neck oncology. Early-stage glottic cancer (T1–T2) can be treated effectively using organ-preserving techniques, such as transoral laser microsurgery (TOLMS) and radiation therapy (RT). Both approaches have shown comparable oncologic outcomes regarding local control and overall survival, but with notable differences in functional results. TOLMS has been associated with slightly higher survival rates and lower recurrence rates, while RT often provides better functional outcomes, especially regarding voice quality and phonation [1,2,3,4].

Despite the effectiveness of these treatments, local recurrence occurs in approximately 10% of early-stage laryngeal cancer cases [5].

The American Society of Clinical Oncology (ASCO) guidelines highlight the importance of salvage strategies for patients who experience treatment failure after RT. While total laryngectomy (TL) is universally considered the gold standard for achieving definitive loco–regional control, it is associated with significant morbidity, including loss of the patient’s voice, an increased risk of pharyngocutaneous fistulas, the presence of a permanent tracheostoma, the need, sometimes, for reconstructive surgical procedures, prolonged hospital stays, and higher management costs [6,7,8].

As such, there has been growing interest in the use of salvage organ-preserving surgeries, particularly open partial horizontal laryngectomy (OPHL), to manage recurrent laryngeal cancer [5,6,7,8,9].

OPHL offers an important alternative to TL, as it can achieve radical oncologic resection while preserving the structural and functional integrity of the larynx. The modular approach of OPHL, introduced by the European Laryngological Society (ELS) in 2014, enables surgeons to tailor the procedure based on intraoperative findings, allowing for a more individualized and potentially less invasive approach to laryngeal cancer treatment [10,11]. This modularity, combined with careful patient selection, has expanded the indications for OPHL in both primary and recurrent laryngeal cancer, providing a viable organ-preserving option, even in cases where previous treatments have failed [12].

Recent data from multi-institutional studies have demonstrated that TOLMS-treated patients, despite the initial success of the organ-preserving approach, present with a recurrence rate ranging from 14% to 16% [13,14]. In these cases, salvage surgery remains a critical intervention. Peretti et al. reported that among 100 patients who experienced recurrence following TOLM, 19 required total laryngectomy (TL), while 7 were successfully treated with OPHL [13]. Similarly, Piazza et al. found that, out of 410 patients analyzed, 59 experienced a recurrence. Of these, 13 patients underwent TL, and 6 were treated with OPHL as a salvage option [14]. These results suggest that OPHL may still serve as a valuable alternative to TL in selected patients, offering satisfactory oncologic outcomes while minimizing the functional deficits associated with TL [9].

This study focuses on a large, multi-institutional cohort of patients who experienced laryngeal cancer recurrence after primary treatment with TOLM. Our aim is to evaluate the efficacy of OPHL as a salvage treatment, exploring its potential to provide both radical oncologic control and preservation of laryngeal function. Additionally, we seek to identify clinical and pathological factors that may predict the risk of second recurrence after salvage OPHL, to optimize patient selection and tailor postoperative surveillance protocols [9,10,11].

The findings of this study will contribute to the growing body of literature supporting the role of OPHL in the multidisciplinary management of recurrent laryngeal cancer and highlight its place as a key strategy in the evolving landscape of organ-preserving oncology treatments.

## 2. Materials and Methods

This study examines a cohort of 66 patients (63 men and 3 women, with an average age of 60.9 ± 12.2 years) who experienced recurrent laryngeal squamous-cell carcinoma (LSCC) following unsuccessful primary TOLMS for glottic cancer (Table 1).

All of these patients underwent a salvage OPHL. The surgeries were conducted at four institutions: Vittorio Veneto Hospital (Treviso, Italy), Martini Hospital (Turin, Italy), Candiolo Cancer Institute (FPO IRCCS—Candiolo, Italy), and the Brazilian National Cancer Institute, between May 1995 and October 2017.

Following the OPHL classification introduced in 2014 by the ELS [10], all procedures were considered conventional in terms of technique and indications and according to the ethical standards of the Institutional and/or National Research Committee and the 1964 Helsinki Declaration and its later amendments. Following national and institutional requirements, ethical review and approval were not required for this study.

The staging of the laryngeal tumors has been carried out or brought back to the 8th edition of the Union for International Cancer Control (UICC) and the American Joint Committee on Cancer (AJCC) staging system [15]. Primary Laser cordectomies were classified according to the 2007 revision of the ELS classification [16].

**Figure 1:**
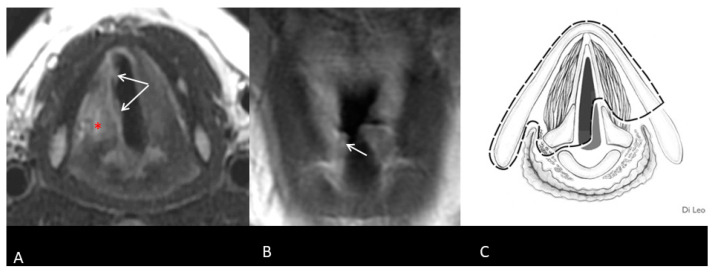
Recurrent right glottic SCC after TOLM: MR sequences FSE T2-weighted on the axial plane (**A**) and FSE T1-weighted after contrast injection on the coronal plane (**B**). An irregular thickening (arrows) of the right side of the glottic level is suspicious for tumor. Oedematous changes are present (asterisk) (**A**). The lesion does not involve the subglottic region (**B**). Axial scheme of resection (Type IIa OPHL + right ARY) (**C**) [10].

**Figure 2:**
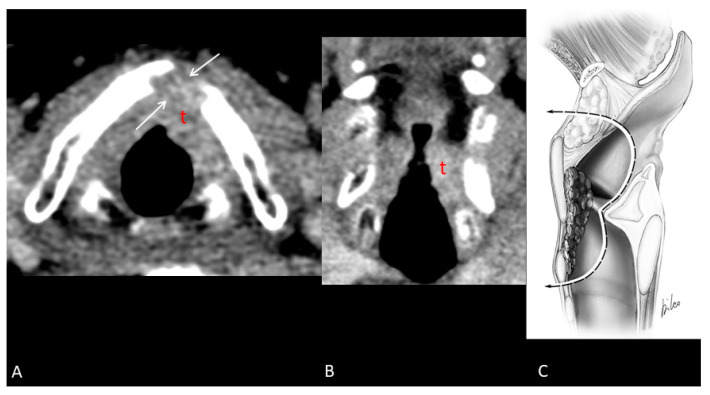
Recurrent glottic SCC after TOLM. Contrast-enhanced CT scan shows neoplastic tissue (t) in the anterior and left sides of glottic level. The lysis (arrows) of the thyroid cartilage is indicative of tumoral invasion at full thickness (**A**). On the coronal reconstruction, the lesion presents transglottic spread; there is no certain tumoral extension into the subglottic region (**B**). Sagittal scheme of resection (Type IIIa OPHL) (**C**) [10].

Before salvage surgery, all patients underwent an in-office examination, biopsy, and imaging of the neck and maxillofacial region, with either MRI or CT scans (Figure 1 and Figure 2). The institutional tumor board then reviewed each patient’s case.

### 2.1. Salvage Open Partial Horizontal Laryngectomies

After informed consent had been obtained, OPHL was employed to manage early glottic recurrences (rcT1-2) and selected cases of intermediate/low volume advanced-stage recurrence (rcT3-4a), as long as at least one cricoarytenoid unit remained functional. In suspect rcT4a cases, there was a minimal extra laryngeal extension, and the patient’s overall health and comorbidities were suitable for surgery.

Based on the ELS classification, the patients underwent OPHL type IIa (supracricoid laryngectomy with cricohyoidoepiglottopexy), type IIb (supracricoid laryngectomy with cricohyoidopexy), type IIIa (supratracheal laryngectomy with tracheohyoidoepiglottopexy) or type IIIb (supratracheal laryngectomy with tracheohyoidopexy). Furthermore, OPHL type II could involve the resection of one arytenoid (Type II + ARY), while type III could be extended to the cricoarytenoid unit (Type III + CAU).

During the salvage OPHL, mucosal surgical margins were routinely examined intraoperatively, using frozen sections. These margins were further reassessed postoperatively in the final pathology report.

### 2.2. Adjuvant Treatments

According to established guidelines, the tumor board evaluated the need for adjuvant radio(chemo)therapy in cases involving extra-laryngeal extension, positive surgical margins, metastatic lymph node involvement classified as higher than pN1, with or without extra-nodal extension, or the presence of other high-risk features noted in the final pathology report.

### 2.3. Follow-Up

The follow-up regimen includes a comprehensive examination every three months during the first two years post treatment, then only a thorough examination every six months from the third to the fifth year, and annually after that. In terms of imaging, a neck MRI or a contrast-enhanced CT scan of the neck and chest is conducted every six months for the first two years and annually from the third to the fifth year. After that, patients are advised to have a neck ultrasound and a chest X-ray once a year.

## 3. Results

A consistent pattern of clinical under-staging has been observed in patients who experienced laser-recurrent laryngeal cancer. Considering only the clinical stage (cTNM), 42 patients (63.6%) with recurrences remained in the early stages at the time of relapse, indicating that 24 patients (36.4%) had progressed to more advanced stages by the time of recurrence.

**Figure 3:**
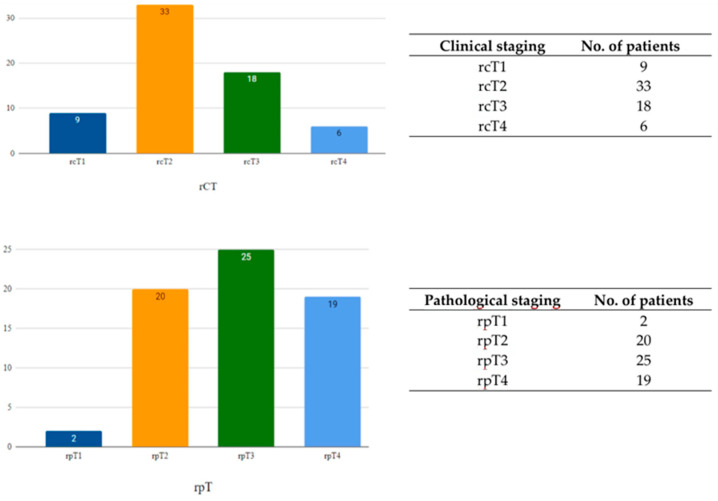
Distribution of patients according to the clinical (rcT) and pathological (rpT) classification of recurrent disease.

Conversely, when pathological classification (pTNM) was considered, only 22 (33.3%) of these patients were still classified as early stage. Regarding T-staging, 44 patients (66.7%) were categorized as stage pT3 or pT4 upon recurrence, with 19 patients (28.7%) already in stage T4 (Figure 3).

Arytenoid mobility, a key factor in assessing laryngeal function, was absent in 15 out of 66 cases, accounting for 22% of patients (Table 2).

In terms of primary procedures, 10 patients (15.2%) underwent type II cordectomy, and 26 (39.4%) underwent type III cordectomy. In comparison, 30 patients (45.4%) underwent subperichondrial cordectomy (type IV: 13, 19.7%; type V: 13, 19.7%, type VI: 4, 6.0%), a more radical procedure reaching and resecting the perichondrium of the thyroid cartilage (Table 3). No patients received planned postoperative radiotherapy.

Regarding the salvage procedures, 47 patients (71.2%) underwent type II OPHL, which involves the resection of the glottis and part or nearly the entire supraglottic region, while preserving at least one cricoarytenoid unit to maintain some laryngeal function. In contrast, 19 patients (28.8%) underwent type III OPHL, a more extensive resection that includes, in addition to supracricoid laryngectomy resection-bloc, part of the cricoid cartilage and one cricoarytenoid unit. This procedure is usually indicated for more advanced tumors (Table 4).

Neck dissection (ND), classified according to the American Academy of Otolaryngology—Head and Neck Surgery Foundation classification, was performed in 52 patients (79%): unilaterally in 41 patients (62%) and bilaterally in 11 patients (16.6%) [17]. Level VI dissection was additionally carried out in 32 patients (48.5%). In six patients (9.1%), the dissection was performed with curative intent for rcN > 0 disease. Conversely, in 14 patients (21.2%) presenting with rcN0 disease, neck dissection was not performed (Table 5).

**Figure 4:**
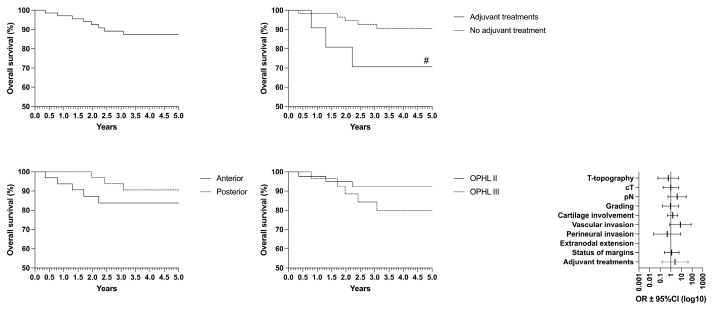
Overall survival (OS) according to the laryngeal compartmentalization, need for adjuvant treatments and type of surgery # = *p* < 0.05 (GBW).

**Figure 5:**
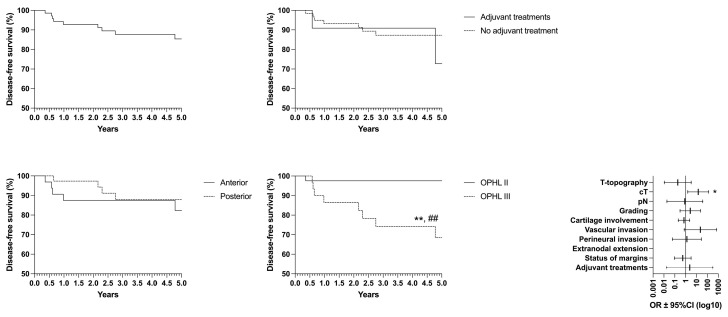
Disease-free survival (DFS) according to the laryngeal compartmentalization, need for adjuvant treatments and type of surgery * = *p* < 0.05, ** = *p* < 0.01 (LR); ## = *p* < 0.01 (GBW).

**Figure 6:**
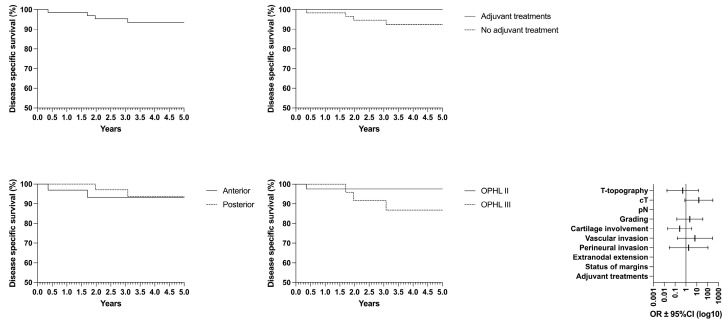
Disease-specific survival (DSS) according to the laryngeal compartmentalization, need for adjuvant treatments and type of surgery.

**Figure 7:**
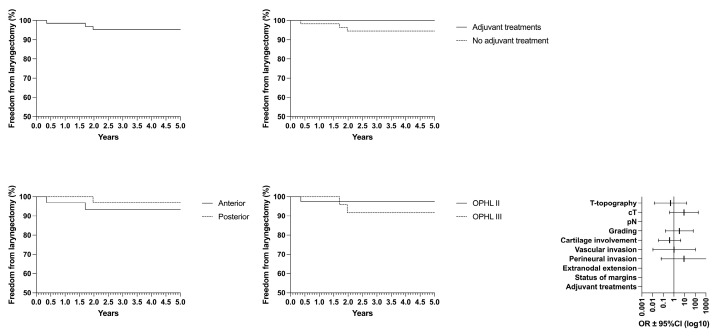
Freedom from laryngectomy (FFL) according to the laryngeal compartmentalization, need for adjuvant treatments and type of surgery.

**Figure 8:**
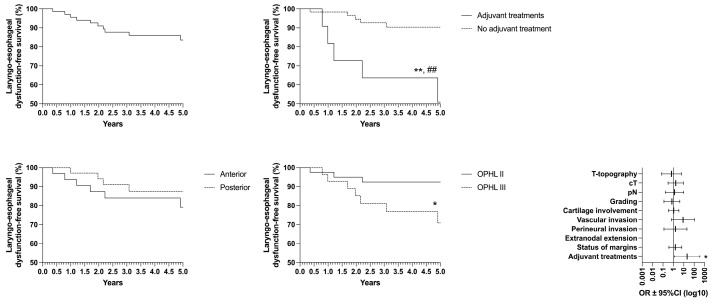
Laryngo–esophageal dysfunction-free survival (LEDFS) according to the laryngeal compartmentalization, need for adjuvant treatments and type of surgery * = *p* < 0.05, ** = *p* < 0.01 (LR); ## = *p* < 0.01 (GBW).

Concerning oncological/functional outcomes, the following results were observed: overall survival (OS) rate of 87.4%, disease-specific survival (DSS) of 93.4%, disease-free survival (DFS) of 85.5%, freedom from laryngectomy (FFL) of 95.3%, and laryngo–esophageal dysfunction-free survival (LEDFS) of 83.6% (Table 6) (Figure 4, Figure 5, Figure 6, Figure 7 and Figure 8).

Pathology reports indicated positive margins in seven cases (10.6%) at definitive histology. Based on pathological findings (gross extra-laryngeal extension, positive margins, multiple pN+, extra-nodal extension), 11 patients (16.7%) were subjected to adjuvant therapy. Seven patients (10.6%) received adjuvant radiotherapy: four patients (6%) with positive margins and extra-laryngeal extension, three patients (4.5%) with pN+. On the contrary, four patients (6%) underwent adjuvant chemoradiation: three patients (4.5%) with pN+ and one patient (1.5%) with extra-nodal extension. Within five years after salvage OPHL, seven patients (10.6%) developed a further locoregional recurrence. Among these, four (6%) underwent further salvage procedures, including total laryngectomy (TL) in three cases (4.5%) and radiotherapy in one case (1.5%). Furthermore, two patients (3.0%) were subsequently treated with chemo-radiotherapy amongst those with total laryngectomy. Univariate analysis revealed that several factors were significantly associated with worse outcomes, including the type of surgery (with type III OPHL being more detrimental), advanced T category, cartilage involvement, poor tumor differentiation (high grade), vascular invasion, perineural invasion, and the presence of Delphian lymph node metastasis. These findings highlight the importance of thorough preoperative evaluation and careful selection of surgical candidates (Table 7). In the multivariate analysis (logistic model), only the yrpT category and vascular invasion emerged as statistically significant independent predictors of recurrence (*p* < 0.05).

Postoperative complications occurred in 7 out of 66 cases (10.6%), but no significant variation was observed in complication rates across different surgical categories. The incidence of sequelae followed a similar pattern, indicating that the type of surgery did not significantly influence the likelihood of long-term complications (Table 8 and Table 9).

## 4. Discussion

In the treatment of early-stage laryngeal carcinoma, salvage interventions play a pivotal role, especially when tumor recurrence is detected early. Early diagnosis allows for the possibility of organ preservation in a significant number of cases, even in the context of recurrent disease. In this regard, bioendoscopy and MRI are crucial diagnostic tools, the latter being particularly important for detecting submucosal recurrences [18,19].

Among the salvage options, open partial horizontal laryngectomy (OPHL) has proven to be a valuable technique, especially after radiation therapy fails. However, robust data regarding its use as a salvage treatment after failed transoral laser microsurgery (TOLM) remain scarce [9,10,11,12,13,14,15,16,17,18,19,20].

A key observation from this multi-institutional series is the upstaging phenomenon, both in clinical and, more notably, pathological restaging, which occurs in recurrent tumors compared to their initial staging. Despite 100% of primary tumors being staged as I-II at diagnosis, 72.7% of recurrences were classified as stage III-IV upon restaging. These findings suggest that a significant number of patients experience disease progression beyond the early stages, raising important questions about the feasibility of performing partial laryngectomy, such as OPHL, in cases of recurrent advanced-stage tumors. This applies even to recurrences after transoral laser surgery, which typically does not compromise the laryngeal framework [13,14,21,22]. The recurrence pattern highlights the complexity of managing these patients and the inherent risks of salvage surgery [9].

This series also shows that many patients initially underwent subligamentous or transmuscular cordectomy, while others had subperichondral resections (30/66, 45.4%). The latter is associated with a higher risk of recurrence, notably when the anterior commissure is involved, which is universally recognized as an adverse prognostic factor for TOLM [14,15,16,17,18,19,20,21,22,23].

In this context, OPHL, with its broader resection capabilities both horizontally (primarily when the arytenoid is resected) and vertically, offers a more radical salvage option than other partial laryngectomies [24,25,26,27].

From a surgical–anatomical perspective, the extent of resection achieved with OPHL type II or III, which are typically recommended for recurrent glottic tumors, should theoretically be sufficient to remove an endolaryngeal recurrent tumor, especially if diagnosed early. Our data support this, as demonstrated by the excellent oncological outcomes in this series, with overall survival (OS) at 87.4%, disease-specific survival (DSS) at 93.5%, and disease-free survival (DFS) at 85.4%.

Interestingly, when cases were stratified by anterior-versus-posterior involvement, according to the laryngeal compartmentalization described by Succo et al., no statistically significant differences in oncological outcomes were observed [28,29]. This might be due to the impact of previous laser surgery, particularly for anterior lesions, which may weaken natural barriers, facilitating extra-laryngeal tumor progression and accounting for the larger extra-laryngeal tumor volume seen in pT4 cases, compared to naïve cases.

Overall survival (OS) was significantly higher in patients who did not require postoperative adjuvant therapy, indicating better long-term survival in those with less extended and aggressive disease. Similarly, disease-free survival (DFS) was notably better in patients who avoided type III OPHL, reinforcing that more extensive surgery is needed in more challenging cases with a higher risk of recurrence. The results are, therefore, particularly favorable when type II OPHL achieves a radical resection (R0), and adverse features such as positive nodes (N+) or extracapsular nodal extension (ENE) are absent. Conversely, freedom from laryngectomy (FFL) remained consistent across all variables, suggesting that the need for total laryngectomy is independent of these factors. However, laryngo–esophageal dysfunction-free survival (LEDFS) was significantly better in patients who did not undergo type III OPHL or require adjuvant therapy. This underscores the functional benefits of less-invasive approaches in select cases [30].

These findings emphasize the importance of careful patient selection for OPHL, particularly in cases with a high risk of understaging at the primary tumor site [31]. Given the risk of clinical cT3 tumors being upstaged to pT4a upon final histological examination, greater caution should be exercised when considering OPHL for high-risk patients. As Crosetti and colleagues have suggested, the indication for OPHL should be limited to clinically staged cT3 cases, as OPHL still provides excellent radicality, even in cases where staging is underestimated (from cT3 to pT4a). However, this is not the case when there is clear clinical evidence of extra-laryngeal extension [32]. In this study, cartilage invasion, vascular invasion, perineural invasion, and involvement of the Delphian lymph node were significant predictors of poor outcomes in univariate analysis, with only the pT stage and vascular invasion remaining significant in multivariate analysis.

From a functional standpoint, salvage OPHL after TOLM does not seem to worsen outcomes compared to OPHL performed on naïve patients, significantly. This contrasts with salvage OPHL after radiotherapy, where functional outcomes are slightly poorer [20,21,22,23,24,25,26,27,28,29,30,31,32,33,34]. However, patients requiring type III OPHL or adjuvant therapy experienced a significant reduction in laryngectomy-free survival (LEDFS), although the overall laryngeal preservation rate was not significantly affected. This suggests that many patients who undergo more extensive surgery, especially when followed by adjuvant therapy, retain their larynx but may struggle with permanent tracheostomy or feeding-tube dependence, due to persistent dysphagia. These findings call for careful consideration when offering this type of salvage surgery to borderline patients.

## 5. Conclusions

In conclusion, data from this large multi-institutional series demonstrate that OPHL is a safe and effective option when applied with the same stringent selection criteria used for naïve patients. The ideal candidates are those with endo–laryngeal lesions, clinically staged as cT3 at most, without multiple clinically positive lymph nodes, and in good general health. In cases with a high risk of understaging (cT3/pT4a), OPHL can still be safely employed, given the broad resection margins, similar to patients not previously treated. However, in patients at high risk of further recurrence following salvage OPHL (cT4a, need for type III OPHL + CAU), strong consideration should be given to total laryngectomy (TL).

Lastly, the altered anatomy following previous laser surgery, particularly regarding natural laryngeal barriers, means that the traditional distinction between anterior and posterior compartmentalization, with or without arytenoid fixation, should not be relied upon as a secure criterion for surgical decision-making. This key point distinguishes post-TOLM salvage surgery from surgery on naïve patients.

In conclusion, this study has some limitations that should be acknowledged. The retrospective nature of data collection introduces potential biases, such as selection bias. Future prospective, randomized trials are necessary to validate our findings. Additionally, incorporating patient-reported quality-of-life metrics post OPHL would offer valuable insights into functional outcomes and patient satisfaction, offering a more comprehensive perspective alongside oncological results.

## Figures and Tables

**Table 1:** Demographic data—66 patients.

		No. of Patients (%)
Age	Mean ± standard deviation	60.9 ± 12.2
	Range	23–79
Gender	Male	63 (95.5%)
	Female	3 (4.5%)
rcT vs. rpT	rcT2-3–>rpT4a	12 (18.2%)
	rcT4–>rpT4a	6 (9.1%)

rcT: clinical classification of recurrent disease; rpT: pathological classification of recurrent disease.

**Table 2:** Arytenoid mobility.

Pathological Staging	Arytenoid Mobility	No. of Patients (%)
pT3	Normal	19 (28.8%)
	Impaired/fixed	10 (15.2%)
pT4	Normal	10 (15.2%)
	Impaired/fixed	5 (7.6%)

**Table 3:** Type of primary CO_2_ laser cordectomy.

Type of CO_2_ Laser Cordectomy	No. of Patients (%)
Type I	0 (0.0%)
Type II	10 (15.2%)
Type III	26 (39.4%)
Type IV	13 (19.7%)
Type V	13 (19.7%)
Type VI	4 (6.0%)

**Table 4:** Surgeries carried out on the 66 patients included in the study.

Type of Treatment (OPHL)	No. of Patients (%)	rcT1	rcT2	rcT3	rcT4
Type IIa	14 (21.2%)	4	8	2	-
Type IIa + ARY	25 (37.9%)	3	19	3	-
Type IIb + ARY	8 (12.1)	1	3	4	-
Type IIIa	2 (3.0%)	-	1	1	-
Type IIIa + CAU	14 (21.2%)	1	1	7	5
Type IIIb + CAU	3 (4.6%)	-	1	1	1

**Table 5:** Neck dissection carried out on the 66 patients included in the study.

Type of Treatment (ND)	No. of Patients (%)
Not performed	14 (21.2%)
Level II–IV unilateral ND	41 (62%)
Level II–IV bilateral ND	11 (16.6%)
Level II–IV unilateral ND + Level VI ND	25 (38%)
Level II–IV bilateral ND + Level VI ND	6 (9.0%)
Level VI ND	1 (1.5%)

**Table 6:** Kaplan–Meier estimates: % (95%CI); GBW: Gehan–Breslow–Wilcoxon test for early events; LR: Log Rank (Mantel–Cox) test for 5-year differences.

	Global	Subcategory		Adjuvant Treatment	
		Anterior	Posterior	Adjuvant	No Adjuvant
OS	87.4 (76.4–93.5)	83.8 (65.2–92.9)	90.6 (73.5–96.9)	70.7 (33.7–89.5)	90.5 (78.6–96.0)
DSS	93.5 (83.4–97.5)	93.3 (75.6–98.3)	93.6 (76.6–98.4)	100	92.4 (80.9–97.1)
DFS	85.4 (73.5–92.2	82.4 (61.7–92.5)	87.9 (70.8–95.3)	72.7 (24.1–93.1)	87.3 (75.0–93.7)
FFL	95.3 (86.2–98.5)	93.4 (76.1–98.3)	97.0 (80.4–99.6)	100	94.6 (84.2–98.2)
LEDfs	83.6 (71.4–90.9)	79.1 (58.5–90.2)	87.5 (69.9–95.2)	50.9 (18.2–76.6)	90.4 (78.3–95.9)
	**Global**	**Surgery**			
		**OPHL II**		**OPHL III**	
OS		92.4 (78.1–97.5)		79.9 (58.0–91.1)	
DSS		97.6 (83.9–99.7)		86.8 (64.4–95.6)	
DFS		97.6 (83.9–99.7)		68.5 (45.8–83.2)	
FFL		97.6 (83.9–99.7)		91.7 (70.6–97.8)	
LEDfs	83.6 (71.4–90.9)	92.4 (78.1–97.5)		70.9 (47.6–85.3)	

**Table 7:** Analyses of factors predicting recurrence.

Variable	Univariable Analysis		Logistic Regression Model	
	Score Test	*p*-Value	Score Test	*p*-Value
Type of surgery	7.779	0.005	3.146	0.076
Tumor site (anterior/posterior)	1.188	0.276	0.057	0.811
Tumor site (glottic/supraglottic)	1.286	0.257	0.717	0.397
pT subcategory	9.474	0.002	5.669	0.017
pN ≥ 1 staging	0.281	0.596	0.086	0.769
Grading	5.457	0.019	2.741	0.098
Cartilage involvement	7.405	0.007	1.691	0.194
Vascular invasion	10.004	0.002	5.104	0.024
Perineural invasion	6.207	0.013	0.031	0.860
Delphian node pN+	8.113	0.004	0.830	0.362
Extranodal extension	0.257	0.612	0.192	0.661
Status of margins	2.747	0.097	0.210	0.647
Adjuvant treatments	0.657	0.418	0.009	0.926

**Table 8:** Complications in the 66 patients included in the study.

Complications	No. of Events (%)	Adjuvant Therapy (N = 11)	Non-Adjuvant Therapy (N = 55)	rcT1 (N = 9)	rcT2 (N = 33)	rcT3 (N = 18)	rcT4 (N = 6)
Cervical bleeding	1 (1.5%)	1 (9.1%)	0 (0.0%)	0 (0.0%)	1 (3.0%)	0 (0.0%)	0 (0.0%)
Kidney failure	0 (0.0%)	0 (0.0%)	0 (0.0%)	0 (0.0%)	0 (0.0%)	0 (0.0%)	0 (0.0%)
Sepsis	1 (1.5%)	1 (9.1%)	0 (0.0%)	0 (0.0%)	1 (3.0%)	0 (0.0%)	0 (0.0%)
Acute myocardial infarction	0 (0.0%)	0 (0.0%)	0 (0.0%)	0 (0.0%)	0 (0.0%)	0 (0.0%)	0 (0.0%)
Respiratory failure	0 (0.0%)	0 (0.0%)	0 (0.0%)	0 (0.0%)	0 (0.0%)	0 (0.0%)	0 (0.0%)
Aspiration pneumonia	4 (6.1%)	1 (9.1%)	3 (5.5%)	1 (11.1%)	1 (3.0%)	2 (11.1%)	0 (0.0%)
Stroke	0 (0.0%)	0 (0.0%)	0 (0.0%)	0 (0.0%)	0 (0.0%)	0 (0.0%)	0 (0.0%)
Wound infection	1 (1.5%)	0 (0.0%)	1 (1.8%)	1 (11.1%)	0 (0.0%)	0 (0.0%)	0 (0.0%)
Fistula	0 (0.0%)	0 (0.0%)	0 (0.0%)	0 (0.0%)	0 (0.0%)	0 (0.0%)	0 (0.0%)
Other	0 (0.0%)	0 (0.0%)	0 (0.0%)	0 (0.0%)	0 (0.0%)	0 (0.0%)	0 (0.0%)
**Total**	7 (10.6%)	3 (27.3%)	4 (7.3%)	2 (22.2%)	3 (9.1%)	2 (11.1%)	0 (0.0%)

**Table 9:** Sequelae in the 66 patients included in the study.

Sequelae	No. of Events (%)	Adjuvant Therapy (N = 11)	Non-Adjuvant Therapy (N = 55)	rcT1 (N = 9)	rcT2 (N = 33)	rcT3 (N = 18)	rcT4 (N = 6)
Tracheostoma stenosis	0 (0.0%)	0 (0.0%)	0 (0.0%)	0 (0.0%)	0 (0.0%)	0 (0.0%)	0 (0.0%)
Laryngeal tight stenosis	4 (6.1%)	0 (0.0%)	4 (7.3%)	1 (11.1%)	2 (6.1%)	1 (5.6%)	0 (0.0%)
Dysphagia	3 (4.5%)	2 (18.2%)	1 (1.8%)	0 (0.0%)	2 (6.1%)	0 (0.0%)	1 (16.7%)
Laryngeal tight stenosis + Tracheostoma stenosis	0 (0.0%)	0 (0.0%)	0 (0.0%)	0 (0.0%)	0 (0.0%)	0 (0.0%)	0 (0.0%)
Dysphagia + Laryngeal tight stenosis	0 (0.0%)	0 (0.0%)	0 (0.0%)	0 (0.0%)	0 (0.0%)	0 (0.0%)	0 (0.0%)
**Total**	7 (10.6%)	2 (18.2%)	5 (9.1%)	1 (11.1%)	4 (12.2%)	1 (5.6%)	1 (16.7%)

## Data Availability

The Authors clarify that, as this study is a retrospective series spanning 20 years, and given the evolving privacy and consent regulations across different national contexts, data sharing outside the individual institutions is not possible. This is because explicit consent for such sharing was not obtained from all patients, some of whom are now deceased.
